# Conflict of interest in Health Technology Assessment decisions: case law in France and impact on reimbursement decisions

**DOI:** 10.3402/jmahp.v3.25682

**Published:** 2015-12-09

**Authors:** Sandrine Frybourg, Cécile Remuzat, Åsa Kornfeld, Mondher Toumi

**Affiliations:** ^a^ Creativ-CeuticalParis, France; ^b^ Faculty of Medicine Public Health Department Research Unit EA 3279, University Aix-Marseille Marseille, France

**Keywords:** conflict of interest, Sunshine Act, Loi Bertrand, HAS, Council of State, HTA

## Abstract

The slow reaction of French authorities to the so-called Mediator® saga in 2009 in France led to investigations that questioned the way conflicts of interest are reported. France implemented the *Loi Bertrand* (‘Bertrand Law’) in May 2013, known as the ‘French Sunshine Act’, with the aim of specifying the scope of disclosure obligations. This policy research reviewed the *Loi Bertrand* and reported case law from the French Council of State (COS) related to conflicts of interest in French Health technology assessment (HTA) opinion. The *Loi Bertrand* requires the publication of most of the agreements concluded between health-care professionals and companies and covers a vast range of health products. Commercial sales agreements of goods and services concluded between manufacturers and health-care professionals are a strong exception to this disclosure obligation. Six cases examined by the COS were analyzed, most of them related to the publication of guidelines or the removal of products from the list of reimbursed drugs and devices. These cases have been reviewed, as well as the impact of the ruling on reimbursement decisions. Four cases led to suspension or invalidation of decisions based on the *Haute Autorité de Santé* (HAS) recommendations due to conflicts of interest. In the two other cases, the HAS provided *post hoc* declarations of interest when required by the COS, and the COS considered the conflicts of interest as irrelevant for the decision. It appears that the COS based its decisions on two main criteria: the acknowledgement of negative conflicts of interest (a link with competitors) and the absence of declarations of conflicts of interest, which have to be presented when required by legal authorities irrespective of when they were completed (even posterior to the HAS opinion). However, the number of cases that have been decided against the HAS remains very limited with respect to the volume of assessments performed yearly. The strengthening of the regulation on declarations of interest might lead to more transparency but also to more cases decided by the COS. A new press investigation (in March 2015) related to alleged cases of conflict of interests led policy makers to amend the Bertrand Law in April 2015 and require the disclosure of amounts paid to health-care professionals by the industry.

The so-called Mediator® saga in France, in which the French authorities were alleged to have reacted slowly to withdraw the diabetes drug Mediator®, led to accusations of conflicts of interest among health-care experts and politicians (1). As a consequence, France passed the Bertrand Law on Strengthening of Health Protection for Medicinal and Health Products in December 2011 (2) so as to enhance the transparency of procedures, among other goals. We will detail and explain this law before we put it into perspective with the recent rulings of the French Council of State (COS), which ruled on several cases reporting conflicts of interest among the French national health agency (*Haute Autorité de Santé* [HAS], the key French health technology assessment organisation). Through a systematic search, we identified six cases that have been ruled on by the COS on this topic. We reviewed these cases, as well as the impact of the ruling on reimbursement decisions.

## Notion of conflict of interest

A widely used definition of *conflict of interest* is ‘a set of circumstances that creates a risk that professional judgment or actions regarding a primary interest will be unduly influenced by a secondary interest’ (3). ‘Primary interest’ refers to the principal goals of the profession or activity, such as the health of patients, the integrity of research, or the duties of public office. ‘Secondary interest’ includes not only financial gain but also motives such as the desire for professional advancement or the wish to favor family and friends. Risks of conflicts of interest have been a concern for a long time: as early as during the seventeenth century, Jean-Baptiste Colbert (one of Louis XIV's ministers) issued an order forbidding ecclesiastics and members of parliament and other commissions to have any activity in the water and forests jurisdictions (4). It should also be mentioned that having multiple interests does not necessarily imply the existence of any conflict of interest. A conflict of interest may arise if these interests are believed to unduly influence decisions.

### The French Sunshine Act

In March 2010, the US Congress passed the Physician Payment Sunshine Act to ‘bring much needed transparency to the financial relationships that exist between the drug and device industries and doctors’ (statement by Senator Charles Grassley) (5). In February 2013, the US authorities published the final regulations, providing further clarification (6). Inspired by the United States, on December 29, 2011, France passed the Bertrand Law on Strengthening of Health Protection for Medicinal and Health Products, also known as the ‘French Sunshine Act’, to establish new transparency procedures for companies producing or marketing health and cosmetic products intended for human usage or providing services associated with such products (2).

The Bertrand Law, named after the former French Health Minister Xavier Bertrand, aimed to restore patient confidence in the health-care system by ensuring transparent, professional, and impartial relationships among health-care providers, pharmaceutical and medical device companies. This law was prepared after the Mediator® saga: the antidiabetes drug Mediator® was withdrawn from the market in late 2009, because it was believed to have caused between 500 and 2000 deaths in France due to serious cardiovascular side effects (7) that many allege could have been prevented. The affair has become politicized because Servier, the pharmaceutical company that manufactured the drug, is reported to have high-level political connections. The health minister himself has been implicated by the French media, who allege that two of his advisors had undisclosed ties to this company (8). Health authorities have been accused of delaying the withdrawal of this drug. However, two assessments of the Transparency Committee (a branch of the HAS that evaluates drugs) on November 17, 1999, and May 10, 2006, recommended withdrawing the product from the reimbursement list because the *service médical rendu* (SMR) (‘actual benefit’) was rated as insufficient due to a negative benefit–risk ratio. Products with an insufficient SMR should not be reimbursed according to French regulation. Despite these assessments, two successive ministers of health decided to maintain the reimbursement status of Mediator® (9).

### Content of the Bertrand Law

After several months of delay due both to the French elections and to intense debates among health professionals, the decree implementing the Bertrand Law No. 2011–2012 was issued on May 21, 2013 (10). This decree aimed at specifying the scope of disclosure obligations, which affect all agreements concluded between health-care professionals and companies, as well as every benefit in kind or in cash representing or exceeding €10 (11).

The decree applies to a vast range of health products under the oversight of the French National Agency of Medicine and Health Products Safety (*Agence Nationale de Sécurité du Médicament et des Produits de Santé*), which is responsible for assessing the safety, efficacy, and quality of health-care products for human use (12–(14))_._ This includes 17 categories of products, such as pharmaceuticals, medical devices, diagnostics, and cosmetics, but also contact lenses and tattoos, among others. The disclosure obligation affects any agreement concluded between companies manufacturing or distributing these products and French health-care professionals (physicians, nurses, ambulance staff, interns, etc.). Such agreements specifically include the following:

- Research and development contracts, e.g., clinical trials and observational studies
- Hospitality at conventions, e.g., invitations to scientific or medical events
- Other consultancy agreements, e.g., speaking positions or positions on advisory boards

With respect to benefits granted to these individuals and entities, enterprises are required to disclose the recipient's identity, the value of the benefit, and the nature and date of the benefit. This law applies to all companies that sell products in France, even those with no subsidiary or registered office located in France.

The only and notable exception to this broad scope is commercial sales agreements of goods and services concluded between manufacturers and health-care professionals. The decree distinguishes between benefit and agreement: an *agreement* is generally defined as ‘a contractual relationship to provide services for or on behalf of the company’. There is no obligation to disclose any amount for commercial sales agreements, which may encompass a wide range of contacts.

On December 19, 2013, the French Ministry of Health published a decree implementing a single website for publication of these disclosures (15). This website only became publicly accessible on June 26, 2014.

### Comparison of the French Sunshine Act and the US Sunshine Act

The French Sunshine Act, while modeled after the US Physician Payments Sunshine Act, is broader in some respects, particularly with regard to the recipients and products covered (16, 17). Whereas the US Sunshine Act only applies to physicians and teaching hospitals, the French law covers benefits provided to those recipients as well as to other health-care professionals (including pharmacists, nurses, and ambulance staff), students in health-care fields, and organizations such as scientific societies and health-care professional associations. Furthermore, the French law applies to manufacturers of virtually all regulated health and cosmetic products, regardless of whether the products are reimbursed under the French social security regime. By contrast, the US Sunshine Act only covers health-care products available under Medicare, Medicaid, or the Children's Health Insurance Program.

### Specific requirements of transparency for health authorities

Before the implementation of this new law, French law already required that members of health authorities report any work, consultant contracts, or any other financial link they may have with any company, association, or other organism active in health care, as stated in the Public Health Code (18). The requirements include an annual declaration of interest disclosure.

The HAS relies on an ethics committee created in 2006 (*Comité Déontologie et Indépendance de l'Expertise*) for the implementation of legislative and regulatory frameworks on the subject of interest (19). The HAS has had a chapter on interests within its ethics charter since 2008 and, in July 2013, its guide on declarations of interests and management of conflicts of interest was updated (20). Upon beginning their duties, all members of commissions or committees, working or advising groups, managerial and supervisory staff, as well as anyone taking part in evaluations or development of guidelines are required to complete a public statement of interests including information about the previous five years, which is available on the HAS website. Interests are also reviewed and discussed before a HAS member takes part in a working group or committee.

In 1993, an antigift law (*Loi Diverses Mesures d'Ordre Social*, or ‘Law on Various Social Measures’) (21) was enacted stating that it was forbidden for health-care professionals to receive any gifts. The law was updated in 2002 to stipulate that the amount of a gift should not exceed €30 (22). In 2008, it was further revised to forbid medical sales representatives from giving any gifts to physicians.

Failure to comply with the Bertrand Law and in particular its guidance on publication of interests may incur a €45,000 penalty for companies and a €30,000 penalty for health-care professionals, as stated in Law No. 2011–2012 (2). The HAS guide on declarations of interests also mentions that anyone taking part in any decision within the HAS while having a conflict of interest may be prosecuted and judged according to Article 432-12 of the Penal Code (€75,000 penalty and five years of imprisonment).

### Ruling of the French COS

Despite these specific requirements on conflicts of interest within the HAS, several legal cases on conflict of interest involving the HAS have occurred, and especially cases ruled by the French COS (*Conseil d’État*), the highest French administrative jurisdiction. It is the final arbiter of cases relating to executive power, local authorities, independent public authorities, public administration agencies, and any other agency invested with public authority (23).

The most famous decision made by the French COS in the field of conflict of interests in health care relates to Formindep's request in 2009. Formindep, a non-profit organization of doctors, went to court after the HAS refused to withdraw two guidelines on type 2 diabetes and Alzheimer's disease (24). Formindep considered that the chairpersons of the groups in charge of the writing of the guidelines had ‘major’ financial conflicts of interest and that four members of the type 2 diabetes working group had failed to file any public statement on conflicts of interest. The COS ruled on April 27, 2011 that the guidelines on type 2 diabetes must be withdrawn because of potential bias and undeclared conflicts of interest among the authors. Indeed, the HAS failed to provide the appropriate documents to the legal authorities when they asked for it. The COS also imposed a fine of €8,000 on the HAS. New guidelines were published two years later.

After the court ruled that the diabetes guidelines must be withdrawn, the HAS withdrew its guidelines on type 2 diabetes on May 2, 2011 and announced on May 18, 2011 that it would also withdraw its Alzheimer's disease guidelines (25). As a consequence, the COS could not rule on this case. The newly appointed HAS chairman announced at the same time that all professional treatment guidelines issued since 2005 would be reviewed for the appropriate management of conflicts of interest and that other guidelines would be withdrawn if necessary. In September 2011, the HAS finally suspended six other guidelines (26):

- Diagnosis, treatment, and monitoring of spondylarthritis (*Diagnostic, prise en charge thérapeutique et suivi des spondylarthrites*, issued in December 2008)
- Vascular disease prevention after cerebral infarction or transient ischemic attack (*Prévention vasculaire après un infarctus cérébral ou un accident ischémique transitoire*, issued in March 2008)
- Rheumatoid arthritis: diagnosis and initial management (*Polyarthrite rhumatoïde: diagnostic et prise en charge initiale*, issued in September 2007)
- Rheumatoid arthritis: management of established disease (*Polyarthrite rhumatoïde: prise en charge en phase d’état*, issued in September 2007)
- Management of complications of breakthrough depressive episodes in the adult population (*Prise en charge des complications évolutives d'un épisode dépressif caractérisé de l'adulte*, issued in April 2007)
- Management of adult patients with high blood pressure (*Prise en charge des patients adultes atteints d'hypertension artérielle essentielle*, issued in July 2005)

Formindep identified further conflicts of interest in HAS guidelines on statins issued in July 2010. According to Formindep, declarations of interests of members of the working group on statins showed strong positive interests with manufacturers of the most expensive branded statins, whereas they had almost no interests with manufacturers whose statin patents had expired (27). Formindep also accused the HAS of accepting declarations of interests of poor quality. To date, the COS has not issued a ruling on this case.

The COS ruled on five other cases, most of them concerning decrees based on Health Ministry decisions to add or remove drugs from the list of reimbursed drugs or other health products from the *Liste des Produits et Prestations Remboursables*, which lists reimbursed medical devices (28–(32)). Most of these decrees were ruled after a technical assessment was performed by the Transparency Commission, a branch of the HAS that publishes drug assessments based notably on the evaluation of the actual benefit (SMR) of the products (33). The SMR assessment is a critical criterion for the decision of the Health Ministry regarding reimbursement.

In three of these five cases, the COS agreed with the claims of the company accusing the HAS of conflicts of interest (29, 30). In the other two cases, the COS found that there was no proven conflict of interest (31, 32)_._

These cases are presented in Table 1.

**Table:** *Table 1*. Cases ruled by the French Council of State against *Haute Autorité de Santé*

Request date	Ruling date	Claimant	Subject	Arguments	Decision		Follow-up

15/02/2006	12/02/2007	Pharmaceutical companies: Top Pharm, Leurquin Mediolanum, Ferlux	Removal of Ribamylase and Megamylase from the reimbursement list	Negative conflict of interest for one member of Transparency Committee (financial interests within a competitor)	Invalidation of the decree + fine of €1000 payable from the State to each company	√	New decree leading to removal of these drugs from the reimbursement list in September 2007
08/11/2011	01/12/2011	Pharmaceutical company: Jolly-Jatel	Removal of Rhinotrophyl from the reimbursement list	Indirect conflict of interest: some members of Transparency Committee had interests with competitors. Urgency required: risk of substitution by therapeutic classes leading to public health risks and higher costs + financial risk for the company as this drug represent 50% of its annual turnover	Suspension of the decree	√	New appraisal by the Transparency Committee in January 2014 leading to a judgment of insufficient actual benefit and advice to remove the drug from the reimbursement list
07/12/2009	27/04/2011	Physicians’ association: Formindep	Publication of guidelines on type 2 diabetes	Four conflict of interest declarations were not available	Abrogation of the guidelines + fine of €8000	√	New recommendations published in 2013
07/12/2009	22/02/2012	Manufacturers of lipolysis devices	Decision to forbid use of five lipolysis techniques for aesthetic purposes	Some declarations of interest of Transparency Committee members were not available Conflict of interest of one member of the Transparency Committee	Request rejected as the HAS provided these documents when required by legal authorities and as there was no proven conflict of interest	X	
19/05/2011	05/09/2012	Pharmaceutical company: Therabel Lucien	Removal of Derinox from the reimbursement list	Conflict of interest of one member of Transparency Committee Urgency required: financial risk for the company as this drug represents a significant portion proportion of its annual turnover + the company is in deficit	Suspension of the decree + fine of €6000	√	New decree leading to removal of these drugs from the reimbursement list in February 2013
16/08/2012	13/11/2013	Pharmaceutical company: Novartis	Refusal to add Exforge Hydrochlorothiazide on the reimbursement list	Conflict of interest of one member of Transparency Committee Two declarations of interest of Transparency Committee members were not available	Request rejected as the HAS provided these documents when required by legal authorities and as there was no proven conflict of interest	X	

*Note*: HAS, *Haute Autorité de Santé* (the French national health agency).

### Categorization of interests/conflicts of interest

These five cases highlight different types of interests and conflicts of interests (34):

- *Direct/indirect conflict of interest*. A conflict of interest may be either direct or indirect. A direct conflict of interest includes engagement in an activity that presents a financial interest or any sign of gratitude that compromises impartial or objective professional standards expected by a member of the health authorities. An indirect conflict of interest relates to a relationship with any person or institution that may have a personal interest and that may influence or compromise the member's professional judgment. This distinction is mentioned in the French law.
- *Positive/negative conflicts of interest*. Positive conflicts of interest imply a direct link between a member of health agencies and a company active in health care, whereas negative conflicts of interests refer to a relationship with a competitor. French law requires only that positive interests be declared upon entry on duty. The decisions of the French COS prove that negative interests have to be taken into account. Indeed, the Jolly-Jatel case led to the suspension of the decree based on the Transparency Committee as some members of the Transparency Committee had interests with competitors of the product evaluated (29). The HAS also mentions the distinction between positive and negative conflicts of interests in its guide on declarations of interests and management of conflicts of interest.
- *Level of interest*. The guide on declarations of interests and management of conflicts of interest, updated by the HAS in July 2013, emphasizes the need to evaluate the level of an interest to discriminate between major interests and other types of interests (20). It states that an interest may lead to conflicts of interest only if it is strong enough to interfere in the fairness of an individual's opinion.
- *Notion of time*. Another key consideration in the evaluation of conflicts of interest is the timing of the submission of a conflict-of-interest declaration. The Formindep case led to the withdrawal of the HAS guidelines because some experts did not provide a declaration (24). Another request made by manufacturers of lipolysis devices was later rejected by the French COS: the claimants stated that several declarations of interest were missing; however the HAS collected these documents *ex post* and provided them when required by the legal authorities, which concluded in September 2012 that there was no conflict of interest (31). Novartis’ request was rejected for the same reasons one year later.

### Criteria leading to a ruling against the HAS and consequences

From the six cases reviewed, it appears that two criteria led the French COS to suspend or invalidate decrees of the Ministry of Health or to abrogate HAS guidelines:

- *A negative conflict of interest*. A proven negative conflict of interest (relationship with a competitor of the product evaluated) seems to be a strong argument to rule against a decision based on HAS recommendations. Negative conflicts of interest are more difficult to detect than positive conflicts of interest. However, according to HAS rules, before taking part in a working group each member is requested to disclose both positive and negative interests.
- *The unavailability of declarations of interest*. The French COS seems to accept a delay in the publication of declaration of interests, as long as these documents are available when required by a judge. Conversely, the inability or the refusal to submit these documents is an argument strong enough to invalidate decisions based on HAS evaluations. These procedures are surprising and could even be considered a challenge to the current rules. The lack of *ex ante* declaration of conflict of interest suggests that HAS procedures need to be strengthened to comply with HAS internal rules. Introducing this requirement into the law may be a good way to secure *ex ante* declaration of interest and to enhance transparency.

It is also interesting to understand the consequences of these decisions from a market access point of view. The HAS rewrote the guidelines a few years after they were withdrawn (following the COS's ruling on type 2 diabetes). As for the proven conflict of interest cases decided by the COS, the Transparency Committee met a few months after these rulings and reached the same conclusions. As a result of these COS decisions, it appears that some companies managed to maintain reimbursement for their products from one and a half to almost three years before these products were once again judged to be of insufficient actual benefit and were definitively excluded from the list of reimbursed products.

## Conclusion

It has not yet been proven whether there was any conflict of interest in the Mediator® case, as the court is still investigating. However, the conclusion that there was a conflict of interest is supported by many judicial investigation reports published in the press. The Transparency Committee concluded twice that there was insufficient SMR, which should have led to the withdrawal of the product from the reimbursement list because of an unacceptable benefit–risk ratio (on November 17, 1999, and May 10, 2006). Therefore, the HAS provided the appropriate recommendation to two different ministers of health and could not be accused of inefficiency or lack of vigilance in this specific case. This saga triggered an extensive law on conflicts of interest, the Bertrand Law, implemented by decree in May 2013.

This topic has also been critical for the COS's ruling against the HAS, as illustrated by the Formindep case with the withdrawal of the diabetes guidelines, followed by the decision of the HAS to withdraw additional guidelines. Out of six cases reviewed by the COS, four decisions were decided against the HAS. It is surprising that the COS considers the *ex post* filing of declaration of interest to be fair. In such a case where the declarations of interest of the members of the Transparency Committee are not disclosed or published, it might be impossible for pharmaceutical or device/diagnostic manufacturers to know whether there is any conflict of interest. Going to court and accusing the HAS of conflicts of interest could result in the pharmaceutical manufacturer paying damages, as the HAS could provide declarations of interest filled out *ex post*. This issue should be further challenged and addressed when amending the Bertrand Law.

It remains to be seen whether the Bertrand Law will help to diminish the number of conflicts of interest. It is noteworthy that all the cases mentioned above predate the decree implementing the Bertrand Law. It is therefore difficult to anticipate whether the French Sunshine Act will address the issues raised by the COS.

Some experts consider the main issue in France to be the lack of in-house experts such as those upon whom the US Food and Drug Administration and other regulatory agencies can rely. Indeed, the risk of this new law is that researchers or experts are discouraged from serving on government advisory committees, as many of them inevitably have industry ties. Another issue is the considerable salary gap between industry and the public agencies. Some experts do not consider the Bertrand Law sufficient to guarantee transparency; for instance, the physicians’ association Formindep filed two requests with the COS in July 2013 (35, 36). Formindep notably protests that details of commercial sales agreements are not included in the declaration of interests, although they can represent significant amounts of money. The differentiation between benefit and commercial sales agreements seems surprising as sales agreements encompass service contracts (37, 38). The French National Medical Council (*Conseil National de l'Ordre des Médecins*) also planned to appeal to the COS on this same subject (39). They commented that ‘it will be possible to know the price of a flight ticket offered to a physician to go to a conference but not the amount that he will get for the presentation he made at this conference’. It seems that there was still some resistance to reaching transparency. For instance, the website designed for the publication of declaration of interest was only made publicly accessible on June 26th, 2014, after Formindep denounced that it was not public.

The French COS issued its ruling on Formindep's requests on February 24, 2015. It ruled that all contracts signed between health-care professionals and the pharmaceutical industry have to be declared and that in particular the amount of these agreements has to be disclosed (unless the contracts relate to the purchase of products for professional activity) (40). Recent allegations by the press that decision makers had conflicts of interest triggered an amendment to the Bertrand Law on April 9, 2015 (41–(43)). The amendment adopted by the parliament (low chamber) requires that the monetary amount of contracts concluded between health-care professionals and the industry (44, 45) be disclosed on the public website www.transparence.sante.gouv.fr.

This new COS case law as well as the decision makers’ conflict of interest reported in the press were therefore an opportunity for policy makers to amend the law and require disclosure of amounts paid to health-care professionals by the industry. However, the need to provide *ex ante* declarations of interests (prior to decisions of the HAS) has still not been highlighted. This is an important element to ensure that conflicts of interest are clearly identified by all commission members before the assessment of a product is initiated. Finally, it is important to put the very small number of cases decided against the HAS into perspective with the number of assessments performed by the HAS. For example, in 2013, the Transparency Committee performed 620 drug assessments and the National committee for the evaluation of medical devices and health technologies (Commission nationale d'évaluation des dispositifs médicaux et des technologies de santé, CNEDiMTS) 163 device or diagnostic assessments (46).

## Conflict of interest and funding

The authors have not received any funding or benefits from industry or elsewhere to conduct this study.
